# Freshwater salinization syndrome limits management efforts to improve water quality

**DOI:** 10.3389/fenvs.2023.1106581

**Published:** 2023-09-22

**Authors:** Carly M. Maas, Sujay S. Kaushal, Megan A. Rippy, Paul M. Mayer, Stanley B. Grant, Ruth R. Shatkay, Joseph T. Malin, Shantanu V. Bhide, Peter Vikesland, Lauren Krauss, Jenna E. Reimer, Alexis M. Yaculak

**Affiliations:** 1 Department of Geology and Earth System Science Interdisciplinary Center, University of Maryland, College Park, MD, United States; 2 Earth System Science Interdisciplinary Center, University of Maryland, College Park, MD, United States; 3 Occoquan Watershed Monitoring Laboratory, The Charles E. Via, Jr. Department of Civil and Environmental Engineering, Virginia Tech, Manassas, VA, United States; 4 Center for Coastal Studies, Virginia Tech, Blacksburg, VA, United States; 5 US Environmental Protection Agency, Center for Public Health and Environmental Assessment, Pacific Ecological Systems Division, Corvallis, OR, United States; 6 The Charles E. Via Jr Department of Civil and Environmental Engineering, Virginia Tech, Blacksburg, VA, United States; 7 Department of Soil, Water, and Ecosystem Sciences, University of Florida, Gainesville, FL, United States; 8 Water Sciences and Policy Graduate Program, University of Delaware, Newark, DE, United States

**Keywords:** freshwater salinization, chemical cocktails, urban streams pollution, riparian buffer, temporal monitoring, longitudinal monitoring, restoration

## Abstract

Freshwater Salinization Syndrome (FSS) refers to groups of biological, physical, and chemical impacts which commonly occur together in response to salinization. FSS can be assessed by the mobilization of chemical mixtures, termed “chemical cocktails”, in watersheds. Currently, we do not know if salinization and mobilization of chemical cocktails along streams can be mitigated or reversed using restoration and conservation strategies. We investigated 1) the formation of chemical cocktails temporally and spatially along streams experiencing different levels of restoration and riparian forest conservation and 2) the potential for attenuation of chemical cocktails and salt ions along flowpaths through conservation and restoration areas. We monitored high-frequency temporal and longitudinal changes in streamwater chemistry in response to different pollution events (*i.e.*, road salt, stormwater runoff, wastewater effluent, and baseflow conditions) and several types of watershed management or conservation efforts in six urban watersheds in the Chesapeake Bay watershed. Principal component analysis (PCA) indicates that chemical cocktails which formed along flowpaths (*i.e.*, permanent reaches of a stream) varied due to pollution events. In response to winter road salt applications, the chemical cocktails were enriched in salts and metals (*e.g.*, Na^+^, Mn, and Cu). During most baseflow and stormflow conditions, chemical cocktails were less enriched in salt ions and trace metals. Downstream attenuation of salt ions occurred during baseflow and stormflow conditions along flowpaths through regional parks, stream-floodplain restorations, and a national park. Conversely, chemical mixtures of salt ions and metals, which formed in response to multiple road salt applications or prolonged road salt exposure, did not show patterns of rapid attenuation downstream. Multiple linear regression was used to investigate variables that influence changes in chemical cocktails along flowpaths. Attenuation and dilution of salt ions and chemical cocktails along stream flowpaths was significantly related to riparian forest buffer width, types of salt pollution, and distance downstream. Although salt ions and chemical cocktails can be attenuated and diluted in response to conservation and restoration efforts at lower concentration ranges, there can be limitations in attenuation during road salt events, particularly if storm drains bypass riparian buffers.

## 1 Introduction

Freshwater salinization increasingly poses risks to aquatic life, drinking water sources, infrastructure, agriculture, and critical ecosystem services (Kaushal et al., 2005; Iglesias, 2020; Kaushal et al., 2021; Cruz et al., 2022; Hintz et al., 2022; Kaushal et al., 2022). Freshwater Salinization Syndrome (FSS) refers to the groups of biological, physical, and chemical impacts which commonly occur together and interact in response to freshwater salinization (Kaushal et al., 2018b; Kaushal et al., 2021; Kaushal et al., 2022). Streams affected by FSS are characterized by elevated concentrations of major ions and trace elements from cation exchange, decreases in biotic richness, increases in eutrophication, and increases in erosion due to sodium dispersion in soils (Canedo-Arguelles et al., 2016; Bird et al., 2018; Kefford, 2018; Behbahani et al., 2021; Kaushal et al., 2021; Szklarek et al., 2022). Global and regional shifts in watershed land use from rural to urban have caused acute and chronic levels of elevated salinity and the potential for the water-quality degradation events *via* FSS (Iglesias, 2020; Kaushal et al., 2022).

Point and nonpoint sources of salt pollution have the potential to release chemical cocktails, which are distinct chemical mixtures of multiple ions and nutrients from a common source that are transported and transformed simultaneously along watershed flowpaths (Kaushal et al., 2018a; Kaushal et al., 2019; Kaushal et al., 2020)^1^. Previous experimental studies in both laboratory settings and field catchments demonstrate how increasing salinization mobilizes one or more elements over time (Amrhein et al., 1992; Bäckström et al., 2004; Kim and Koretsky, 2013; Duan and Kaushal, 2015; Kaushal et al., 2018a; Haq et al., 2018; Kaushal et al., 2019; Galella et al., 2021; Kaushal et al., 2022). Distinct and heterogeneous chemical mixtures occur along affected waterways and reflect different pollution sources, road salt events, or biogeochemical transformations. For example, elevated concentrations of Na^+^, Cl^−^, SO_4_^2−^, and K^+^ can suggest a chemical cocktail of point source sewage and wastewater effluent discharge (Rose, 2007). Road salt events mobilize multiple contaminants into solution, including base cations, trace metals, nutrients, and organic matter (Rhodes et al., 2001; Bäckström et al., 2003; Bäckström et al., 2004; Green et al., 2008; Rodrigues et al., 2010; Haq et al., 2018). Road salt pulses can mobilize chemical cocktails of cations (*e.g.*, Ca^2+^, Na^+^, and Mg^2+^), heavy metals (*e.g.*, Cu, Sr^2+^, and Mn), and NH_4_^+^ from soil particles via soil cation exchange and other complicated dynamics (Amrhein et al., 1992; Bäckström et al., 2003; Bäckström et al., 2004; Green and Cresser, 2008; Findlay and Kelly, 2011; Kaushal et al., 2019; Galella et al., 2021; Kaushal et al., 2021). Stormwater runoff, another urban issue that degrades the water quality in streams, can have a chemical cocktail of Na^+^, Cl^−^, HCO_3_^−^, Ca^2+^, Mg^2+^, and Sr^2+^ from the weathering of impervious surfaces and other sources (Kaushal et al., 2017; Masoner et al., 2019). Classifying groups of chemical cocktails based on elemental combinations can facilitate tracking the fate and transport of different pollution sources along flowpaths in the field and at a watershed scale.

The general composition of the salt-ion enriched chemical cocktail is influenced by the influx of salt ions and the mobilization of trace metals in sediments and soils (Findlay and Kelly, 2011; Galella et al., 2021). The dominant cation of road salt, Na^+^, has a large radius and a high charge with respect to the other monovalent cations (Findlay and Kelly, 2011). Therefore, Na^+^ is very effective at competing for negatively charged sites on clay soil particles and displaces other adsorbed ions into solution, including Ca^2+^, K^+^, Mg^2+^, Pb, Cu, Mn, and Sr^2+^ (Amrhein et al., 1992; Findlay and Kelly, 2011; Galella et al., 2021). Salinization can also mobilize total dissolved nitrogen, dissolved organic carbon, and protein-like fluorophores into solution (Amrhein et al., 1992; Kaushal et al., 2022). Detailed road salt event monitoring over time has shown there are possibly two types of chemical cocktails which form during road salt events: the first pulse of Na^+^ from initial application (Kaushal et al., 2017; 2019; Galella et al., 2021) and a subsequent peak of other ions, nutrients, metals and organics due to ion exchange, dispersion of soils, and other processes (Kaushal et al., 2019; Galella et al., 2021; Kaushal et al., 2022; Kaushal et al., 2023b; Galella et al., 2023). By studying changes in the composition of chemical groups longitudinally along a flowpath (Kaushal et al., 2023a), we can better assess the transport, transformation, and attenuation capacity of salt ions and associated contaminants (*i.e.*, the chemical cocktail) mobilized by freshwater salinization (Kaushal et al., 2018b; Kaushal et al., 2023b).

Although an increase in the scope and magnitude of FSS has been documented over time (Rodrigues et al., 2010; Bird et al., 2018; Kaushal et al., 2018b; Bhide et al., 2021; Kaushal et al., 2021; Grant et al., 2022; Kaushal et al., 2023b), the natural capacity of watershed flowpaths to retain or dilute chemical cocktails mobilized by freshwater salinization longitudinally are often less studied (Maas et al., 2021). Here, “flowpath” refers to the permanent, in-stream route in which water follows within a drainage basin. It is also unknown whether the attenuation capacity of a flowpath can be exceeded or saturated under different environmental conditions, such as different pollution events (Maas et al., 2021; Grant et al., 2022; Kaushal et al., 2022; Galella et al., 2023). Some salt ions from freshwater salinization, such as Na^+^ and Cl^−^ can be attenuated along watershed flowpaths through ion exchange and dilution (Maas et al., 2021; Kaushal et al., 2022; Kaushal et al., 2023a; Galella et al., 2023). However, there are uncertainties in the distance and extent of the water quality impacts of freshwater salinization transmitted downstream.

Currently, we do not know if mobilization of chemical mixtures can be mitigated or reversed in any way using restoration and conservation. Although conservation and restoration can be broadly defined, there are potential changes in water quality that can be explored. These areas can serve as “hot spots” of biogeochemical transformations, primarily through denitrification and nutrient removal in the hyporheic zone of the floodplain; but also can retain multiple ions and pollutants to sediments, among other processes (Mayer et al., 2007; Vidon et al., 2010; Kaushal et al., 2020). However, the effects of restoration and stormwater management strategies on the attenuation of salt pollution sources and chemical mixtures require further investigation (Snodgrass et al., 2017; Maas et al., 2021; Kaushal et al., 2022; Kaushal et al., 2023a; Galella et al., 2023). Restored floodplains can possibly attenuate salt ions along flowpaths, but there can also be the potential for contaminant mobilization due to ion exchange, changes in pH and solubility, and stimulation of microbial processes (Kaushal et al., 2022; Kaushal et al., 2023a; Kaushal et al., 2023b; Galella et al., 2023). Whether conservation and restoration efforts can attenuate salt pollution sources, similarly to other chemical pollutants, remains an important question in water quality studies (Maas et al., 2021).

In this paper, we investigate how chemical cocktails mobilized by freshwater salinization are transmitted or attenuated along watershed flowpaths, across different pollution events, and in response to conservation and restoration efforts. Our hypothesis is that there are changes in longitudinal concentrations across flowpaths transitioning into different land uses and seasons; nonpoint and point sources of pollution in streams will result in different longitudinal and seasonal patterns. Whereas most work has focused on understanding changes in salinization over time, our analysis highlights potential retention and release pathways of chemical mixtures along watershed flowpaths, including through restoration and conservation areas. We investigate the changes in the concentrations and chemical cocktails in response to three potential sources of salinization pollution: wastewater treatment plant effluent, stormwater runoff, and road salt application; all of these are common forms of pollution in the Mid-Atlantic region of the United States and other regions. Our results can also help identify seasonal and environmental conditions under which chemical mixtures that are enhanced by salt pollution (chemical cocktails) can be formed, transported, or attenuated in stream water.

## 2 Methods and site descriptions

### 2.1 Study design: monitoring FSS along watershed flowpaths across time and space

**FIGURE 1:**
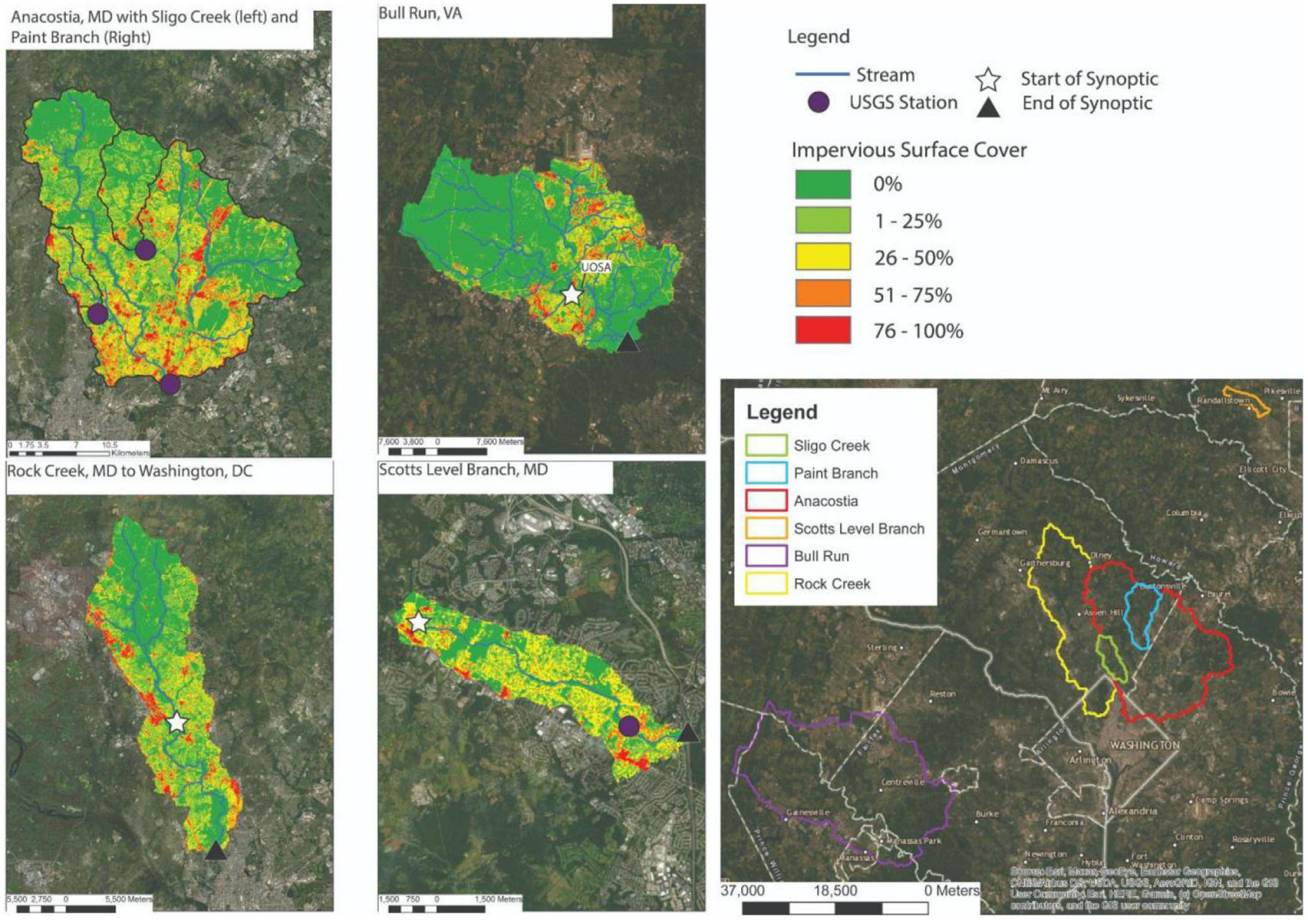
Impervious surface cover and sampling sites for the temporal and spatial monitoring of study watersheds in the Mid-Atlantic region of the United States. White stars indicate the start of the spatial monitoring and black triangles indicate the end of the spatial monitoring. Purple circles indicate the USGS gauging stations monitored during the study, three within the Anacostia watershed and one within the Scotts Level Branch watershed.

Six watersheds located in Maryland (MD), Washington D.C., and Virginia (VA) were monitored spatially and temporally along the watershed flowpath (Figure 1). Five of the six stream sites have U.S. Geological Survey (USGS) gauges with high-frequency sensors (Table 1). Four of these sites were sampled every 2 weeks (Sligo Creek, Paint Branch, Scotts Level Branch, and Anacostia River) to assess the development of chemical cocktails under various seasonal and weather conditions. Three streams (Scotts Level Branch, Rock Creek, and Bull Run) were sampled longitudinally once a season to characterize spatial changes in water chemistry, evaluate the potential effects of restoration and conservation areas, and chemical cocktail formation or attenuation along the flowpath. These three streams also have long-term monitoring sites on the flowpath. The two sampling regimes employed in our study allowed us to explore whether there was a difference in the formation and attenuation of chemical cocktails mobilized by freshwater salinization over a range of weather conditions, seasons, land uses, land management practices, and pollution events.

### 2.2 Temporal monitoring of water quality and site descriptions

Between 08 September 2021 and 23 September 2022, samples were collected twice a month at four U.S. Geological Survey gauging stations to analyze stream chemistry over an annual cycle. These sites are: the Anacostia River at Bladensburg Waterfront Park (USGS 01651007), Scotts Level Branch at Rockdale, MD (USGS 01589290), Sligo Creek near Takoma Park, MD (USGS 01650800), and Paint Branch near College Park, MD (USGS 01649190) (Table 1) and briefly described below. In addition to stream samples, high-frequency data for specific conductivity (SC, in micro-Siemens per centimeter at 25°C), discharge (Q, in cubic feet per second), pH, and temperature (T in °C) were collected from the USGS National Water Information System (U.S. Geological Survey, 2019b); however the USGS gauge at Anacostia River at Bladensburg Waterfront Park did not have discharge recorded at the gauge. The discharge and specific conductance for the temporal monitoring are shown in SI Supplementary Figure S1. Precipitation for the region varied by season (Supplementary Figure S2) and in the Mid-Atlantic region, annual snowfall is 22.0 inches and the maximum snowfall is 73.2 inches (Dulles VA Snowfall, 2022). The sampling sites are described below.

The Anacostia River is one of the largest tributaries of the Potomac River and drains parts of Maryland and Washington, D.C. (Velinsky et al., 2011). In this study, three of the temporal monitoring locations lie within the Anacostia Watershed. Paint Branch and Sligo Creek are two tributaries to the Anacostia River. The sampling site on the mainstem of the Anacostia at Bladensburg is freshwater tidal with a semidiurnal tide up to 1 m in height (Velinsky et al., 2011). Biological, nutrient, sediment, and bacterial impairment and degradation of the Anacostia caused by regional urbanization has occurred over the past 200 years (Velinsky et al., 2011; Miller et al., 2013). The Anacostia at Bladensburg site has the largest drainage area of the sites sampled here, the shortest USGS gauge record, and does not have discharge recorded (Table 1).

Sligo Creek is a highly urbanized tributary to the Anacostia River near Takoma Park, Maryland. The sampling site on Sligo Creek is next to the USGS gauge and downstream of numerous stream restorations, including stormwater retrofits, constructed wetlands, channel reconstruction, tree planting, and fish stocking projects implemented in 2001 (Stranko et al., 2012).

Paint Branch, located near College Park, Maryland, is a tributary of the Northeast Branch of the Anacostia River. Due to military site restrictions, we sampled 1.5 km upstream of the USGS gauge, which is located between Montgomery and Prince George’s County (Miller et al., 2013).

Scotts Level Branch is in Randallstown, Maryland near Baltimore, Maryland, and has the smallest drainage area of the sites sampled. The drainage area is residentially developed, with two floodplain reconnection restoration sites along the flowpath (Scotts Level Branch Stream Restoration Project, 2019).

### 2.3 Longitudinal monitoring of stream flowpaths across land use

The relationship between chemical cocktails associated with FSS and varying watershed characteristics was investigated with particular attention to watershed management features. Three streams, Bull Run, Rock Creek, and Scotts Level Branch were sampled along the flowpath seasonally. Each has a long-term streamflow and water quality monitoring network stations. There are different types of watershed management features along the flowpath at each of the streams, including regional parks, a national park, and stream-floodplain reconnection. Each stream was studied during baseflow and two road salt events. The sampling conditions during each road salt synoptic is outlined in Supplementary Figure S3. Brief descriptions of synoptic sampling sites along stream flowpaths are summarized below.

#### 2.3.1 Bull Run (flowpath from upstream of a wastewater treatment plant through a regional park)

Bull Run flows through four regional parks: Manassas National Battlefield Park (20 km^2^), Johnny Moore Stream Valley Park (less than 0.005 km^2^), Bull Run Regional Park (6 km^2^), and Hemlock Overlook Regional Park (1.6 km^2^) (Manassas, 2021; Hemlock Overlook Regional Park, 2022; Johnny Moore Stream Valley Park, 2022; Northern Virginia Regional Park Authority, 2022). Synoptic sampling began at Bull Run Regional Park and Hemlock Overlook Regional Park. The confluence of Bull Run and a rapidly urbanizing tributary, Cub Run, occurs within Bull Run Regional Park.

The Upper Occoquan Service Authority (UOSA) operates a water reclamation facility which discharges 54 million gallons of treated wastewater effluent per day into Bull Run within Bull Run Regional Park (Onyango et al., 2014). The discharge flows to the Occoquan Reservoir and is part of an indirect potable reuse (IPR) system (Cubas et al., 2014). The UOSA treatment process includes a conventional treatment, chemical and physical advanced treatment, disinfection, and digestion and sludge handling before discharging to Bull Run (Upper Occoquan Service Authority, 2022). The wastewater treatment process can remove biological nitrogen, therefore discharging reclaimed water with lower phosphorus and organic matter concentrations (Cubas et al., 2014). Nitrified effluent is discharged to Bull Run in the warmer months to combat the start of anoxic conditions in the reservoir; in the winter months when the reservoir is oxygenated, UOSA must remove nitrogen in the wastewater and follow regulations for point source nitrogen loads (Cubas et al., 2014). UOSA is the only wastewater treatment plant within the studied watersheds.

Four longitudinal synoptic surveys were conducted along the length of Bull Run. Each synoptic included portions of Bull Run above and below the location where the treated wastewater is discharged from UOSA. The first two synoptic sampling events were conducted during baseflow conditions and extended from Bull Run Regional Park to the Occoquan Reservoir. The last two synoptics were sampled during snow events and at a high-frequency along the flowpath (Table 2, Supplementary Figure S3); we were not able to sample the full length of the previous synoptics due to safety concerns.

#### 2.3.2 Rock Creek (flowpath from urban land use through a forested national park)

Rock Creek flows through Rock Creek Regional Park in Maryland and directly into Rock Creek National Park in Washington, D.C. Rock Creek Regional Park, at about 7.3 km^2^, is managed by the Maryland-National Capital Park and Planning Commission (Montgomery Parks, 2022). Downstream of the regional park, the 7.1 km^2^ Rock Creek National Park in Washington D.C. was established in 1890 by the National Parks Service (National Parks Service, 2022). While the river flows through two geographical regions, about 80% of Rock Creek’s drainage area is in Maryland and the remaining 20% in D.C. (Cintron, 2016). Rock Creek has been the focus of numerous water quality studies, addressing the contribution of the stormwater drain runoff and the 217 combined sewer outfalls to pathogen, metal, and organic matter pollution in the stream (Miller et al., 2013; Cintron, 2016).

Five longitudinal synoptic surveys were conducted along Rock Creek, each with varying distances downstream. All synoptics began in Rock Creek Regional Park in Maryland and ended in Rock Creek National Park in Washington, D.C. (Table 2). Road salt event 1 was sampled 4 days after a snow event and the second road salt event was collected 3 days after a smaller snowstorm (Supplementary Figure S3).

#### 2.3.3 Scotts Level Branch (flowpath through suburban land use with floodplain reconnection)

Scotts Level Branch is a suburban stream with a narrow riparian buffer (Wood et al., 2022). Two floodplain reconnection projects were completed on Scotts Level Branch (Scotts Level Branch Stream Restoration Project, 2019). The first restoration project at McDonogh Road was completed in the spring of 2014. Around 2,000 linear feet of the stream was restored, starting 2.6 km downstream of the headwaters (Scotts Level Branch Stream Restoration Project, 2019). The second project began in the summer of 2019 at the headwaters, a large storm drain, near Marriottsville Road and extended 1,500 feet to Tiverton Road (Scotts Level Branch Stream Restoration Project, 2019). Unlike at Rock Creek and Bull Run, sampling at Scotts Level Branch was conducted at the same location during each synoptic. Scotts Level Branch was the shortest stream length with restricted access due to the residential areas, therefore, we could only access certain locations. Samples were collected from the headwaters of Scotts Level Branch originating at a stormwater drain to the confluence of Scotts Level Branch and the Gwynns Falls.

### 2.4 Sample processing and elemental analyses of water samples

All water samples were collected in 125 mL sample bottles that were rinsed in stream water three times prior to sample collection. A Yellow Springs Instrument (YSI) ProQuatro multiparameter meter was used to collect *in situ* data for temperature, conductivity, pH, dissolved oxygen, and oxidation-reduction potential (ORP). Samples were collected along the mainstem and in the tributaries of the streams when possible. Samples were collected 100 m downstream of the confluence of a tributary and a stream to ensure the sample was well mixed and representative of the stream (Kaushal et al., 2014; 2023a).

Laboratory methods for sample processing and elemental analyses follow those described previously (standard methods, for example, see Haq et al., 2018; Kaushal et al., 2021; Wood et al., 2022). Each water sample was filtered through an ashed 0.7-μm glass fiber filter. Unacidified samples were analyzed on a Total Organic Carbon Analyzer (TOC-L, Shimadzu, Columbia, MD, USA) for total dissolved nitrogen (TDN). The detection limit for total dissolved nitrogen is 5 μg/mL with a maximum reproducibility coefficient of variance of 1.5% (Shimadzu, 2011). An aliquot of the filtered sample was acidified to 0.5% using ultra-pure nitric acid to prevent flocculation and biological activity. Acidified samples were analyzed via inductively coupled plasma optical emissions spectrometry (ICP-OES) using a Shimadzu Elemental Spectrometer (ICPE-9800, Shimadzu, Columbia, Maryland, USA). The elements analyzed include B, Ba^2+^, Ca^2+^, Cu, Fe, K^+^, Mg^2+^, Mn, Na^+^, S^2−^, Sr^2+^. The limits of detection for the TDN and cations are indicated in Supplementary Table S1 (Shimadzu, 2023).

### 2.5 Land use characterization along stream flowpaths

Watershed characteristics, including the percent forest cover, the percent impervious surface cover, and drainage area were obtained from a 30 m resolution dataset from the 2019 National Land Cover Database using the USGS StreamStats web application and ArcMap 10.4 (U.S. Geological Survey, 2019a). Cover and drainage area estimates were determined at each sampling location along the stream, allowing water quality measurements and watershed characteristics to be coupled. Approximate estimates of riparian buffer width (km) were determined at each sampling location using 2022 satellite images in Google Earth. Riparian buffer width was defined as the maximum distance between natural land cover (forest or shrubland) on the left stream bank and the right. Distances were always measured perpendicular to the stream’s flowpath for analytical consistency. Riparian buffer width differs from percent forested cover in that it only reflects the width of the vegetated zone immediately adjacent to each sampling location (*i.e.*, it is a localized measure). Forested cover reflects the broader drainage area at each sampling location, which is broader in scope.

Significant tributaries along each stream were identified using USGS StreamStats. At Rock Creek and Bull Run, any stream that was named or could be visually identified using StreamStats was considered a major tributary. At Scotts Level Branch, any second order stream identified via StreamStats was considered a major tributary.

### 2.6 Statistical analyses

Principal component analysis (PCA) was used to investigate dominant temporal and spatial parameters across multiple analytes (*sensu* Helena et al., 2000; Ouyang, 2005; Hu et al., 2013). PCA was performed three ways. First, all ion data collected at streams that were sampled bi-monthly (Section 2.2) were pooled, and PCA was performed on the collective data. The focus of this analysis was on characterizing the different types of chemical cocktails that are present at across different seasons and times. The second PCA focused on all sites that were sampled longitudinally (Section 2.3) and elucidating common changes in chemical cocktails along their flowpaths. The final PCA had this same longitudinal focus, but was performed on a site-by-site basis (*i.e.*, separately at Bull Run, Rock Creek, and Scotts Level Branch) so that any elucidated patterns in the transformation or attenuation of chemical cocktails would be site-specific.

Prior to performing PCA, data were evaluated for normality using histograms and quantile-quantile plots and determined to be positively skewed. Given this, all variables were log-transformed to avoid violating normality constraints (Davis, 2002). Following transformation, data were standardized to have a mean of zero and a standard deviation of one, such that our final principal components represent the eigenvectors of our data correlation matrix. PCA was conducted in R studio version 1.4 using FactoMineR and factoextra (Lê et al., 2008). A resampling-based stopping rule analogous to the Rand-Lamda rule defined by Peres-Neto et al. (2005) was used to identify principal components that explained significantly more variance than expected due to chance at a *p* < 0.05 level (Rippy et al., 2017). Only these components were retained and interpreted in our analysis.

Multiple linear regression (R package glmulti; Calcagno and Mazancourt, 2010) was used to determine if landscape characteristics (*e.g.*, drainage area, impervious cover, forest cover, riparian buffer width; calculated as described in Section 2.5) were significant drivers of longitudinal variability in within-stream chemical cocktails characterized using PCA. Two different MLR models were developed, one for Rock Creek, and one for Bull Run. Scotts Level Branch was not evaluated given the limited spatial coverage at this site. For each of these models, the dominant chemical cocktail (*i.e.*, the first principal component across all evaluated ions) was the dependent variable, and landscape characteristics (see list above) were possible independent variables. An additional independent variable “Sampling Event” was also evaluated to account for variability in chemical cocktail composition over time; such variability is expected to have myriad underlying causes that are not possible to resolve here given the limited number of synoptics conducted.

Prior to performing MLR, the variance inflation factor was calculated across all independent variables to check for multicollinearity. Impervious surface cover was found to be collinear with forest cover at Rock Creek (inflation factor >5; Zuur et al., 2010) and was not evaluated further. At Bull Run, impervious surface cover and forest cover were both collinear with drainage area, resulting in their exclusion from MLR analyses for this stream. All possible combinations of the remaining independent variables were fit and the resultant MLR models were ranked using the Bayesian information Criterion (BIC; Schwarz, 1978). The best fit model family (*i.e.*, models within 2 BIC units of the highest ranked model) was identified and screened for predictive ability using leave-one-out cross validation with Root Mean Squared Error (RMSE) as the validation metric (see Hawkins et al., 2003; Parker et al., 2017). For each stream, the model with the lowest RMSE (averaged across all cross-validation estimates) was selected as the best-fit model. BIC weights were estimated as in Wagenmakers and Farrell (2004) to determine the relative likelihood that each best-fit model is truly best given the candidate models evaluated. The coefficient of determination (*R*^2^) of each best-fit model was estimated as a measure of overall model fit. Subsequently, averaging over ordering (R-package relaimpo; Groemping, 2007) was used to estimate the proportional contribution of each independent variable to these *R*^2^ estimates. This provides a measure of the relative importance of each independent variable to in-stream chemical cocktails.

## 3 Results

### 3.1 Stream water chemistry changes temporally along a land use gradient

**FIGURE 2:**
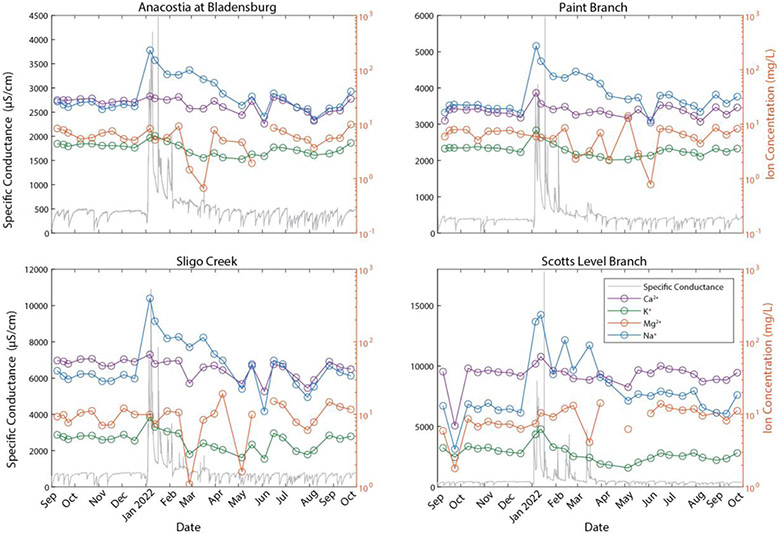
Time series of the temporal monitoring sites and the base cations concentrations (mg/L) plotted with respect to specific conductance. Samples were collected during September 2021–September 2022. The synchronous peak in specific conductance and the ions was due to road salt application.

**FIGURE 3:**
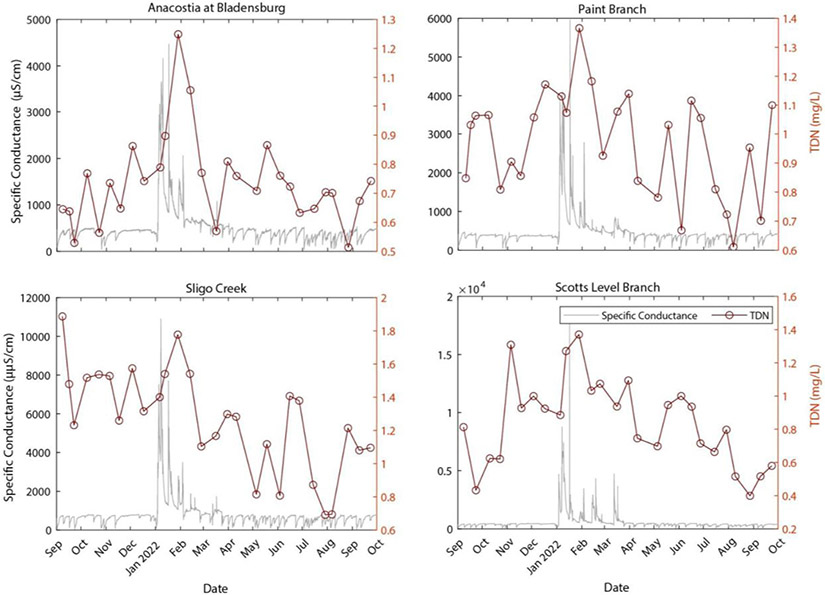
Time series of total dissolved nitrogen (mg/L) with respect to specific conductance.

Over an annual cycle, there were seasonal changes in water quality in the salt ions and nutrients (Figures 2, 3) at the four bi-monthly sampling sites. Specific conductance and salt ion concentrations, such as Na^+^, K^+^, and Ca^2+^, peaked sharply during winter months likely due to road salt applications (Figure 2). There was seasonal variability in the total dissolved nitrogen (TDN) concentration, with the highest TDN concentrations following wintertime peaks in specific conductance.

### 3.2 Stream water chemical cocktails form seasonally and during different FSS pollution events

#### 3.2.1 Chemical cocktails are formed seasonally over an annual cycle

**FIGURE 4:**
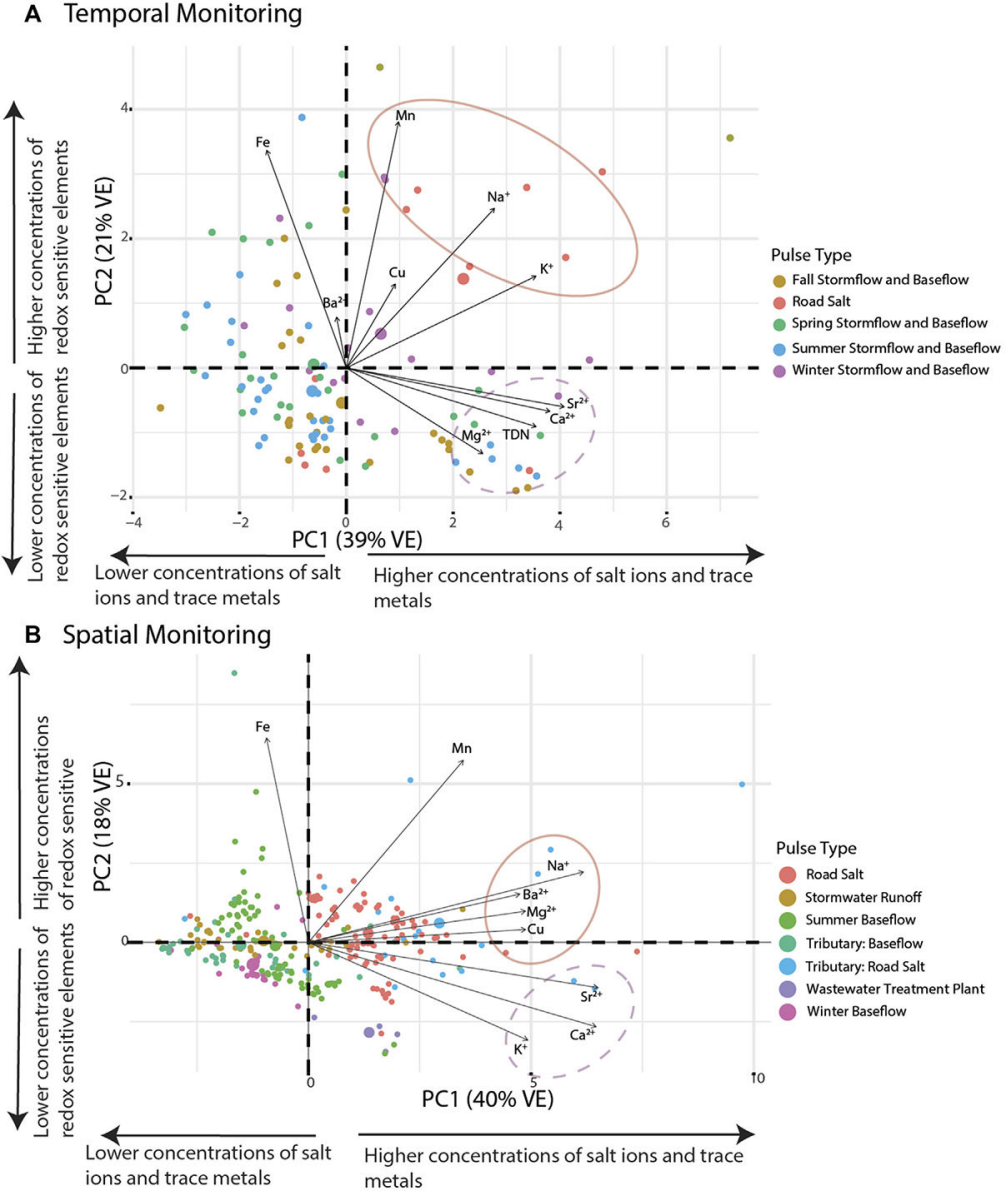
Principal component analysis of different sources of pollution. There is a clustering of salt ions based on the pollution event. VE indicates the amount of variance explained by each principal component. **(A)** includes samples collected temporally from the Anacostia, Paint Branch, Scotts Level Branch, and Sligo Creek and **(B)** includes samples collected spatially from Bull Run, Rock Creek, and Scotts Level Branch. The road salt events dominate the PCA on the right-hand side of the plot, as indicated by the circled ions Na^+^, Ba^2+^, Mg^2+^, and Cu. The dashed line is a chemical cocktail that can be indicative of urban weathering, composed of K^+^, Ca^2+^, and Sr^2+^.

The relationship between specific conductance and multiple ion mobilization suggests that chemical cocktails associated with salinization are formed in urban streams (*e.g.*, Kaushal et al., 2018a; Kaushal et al., 2019; Morel et al., 2020; Galella et al., 2021). PCA was used to diagnose the composition of the chemical cocktails which formed seasonally over an annual monitoring cycle (Figures 2, 3). Dimension 1 of the PCA (PC1) (Figure 4, see Supplementary Table S2 for the discriminant matrix) captures patterns in salt ions and trace metals (~40% variance explained), whereas dimension 2 of the PCA (PC2) captures patterns in redox sensitive elements (~21% of the variance explained). Water samples collected during road salt pulses are characterized by elevated concentrations of Na^+^, K^+^, Cu, and Mn (see pink circles in the top right quadrant of Figure 4A; small symbols indicate the individual samples collected, large symbols indicate their central tendency). Most of these ions, except for Cu, have a positive significant relationship with increasing specific conductance (Supplementary Table S2). Water samples collected during summer baseflow and stormwater events had lower concentrations of Na^+^, K^+^, Cu, and Mn (see blue symbols loading opposite the road salt cluster, Figure 4A).

A second chemical cocktail can be seen in the lower right quadrant of Figure 4A, characterized by elevated concentrations of Ca^2+^, Mg^2+^, Sr^2+^, and TDN. These elements had a positive significant relationship with increasing impervious surface cover (Supplementary Table S2). Stormflow and baseflow samples collected in the fall (brown symbols), spring (green symbols), and summer (blue symbols) had variable concentrations of constituents in these chemical cocktails (note the variability in the locations of samples from these groups with respect to PC1). Only baseflow and stormflow samples collected in the winter exhibited chemical cocktails that were similar to those evident during road salt events. Notably, you can trace a cycle from one chemical cocktail towards the next by following the seasons, starting with road salt in the upper right quadrant, shifting down and left (away from chemical cocktail 1 as winter transitions to spring and then to summer), relaxing towards chemical cocktail 2 (lower right quadrant) transitioning into fall. The stark differences between chemical cocktails by season illustrate the importance of factoring seasonal changes into our understanding of the mobilization of chemical cocktails by freshwater salinization. Moreover, the relationship between different chemical cocktails and variables like land cover (imperviousness) or management practices (road salt application) highlight the importance of these factors of seasonal change.

#### 3.2.2 Chemical cocktails are formed spatially across different FSS pollution events

Chemical cocktails formed longitudinally following different pollution pulse events. This is evident in Figure 4B, where water samples from the longitudinal sampling events at Bull Run, Rock Creek, and Scotts Level Branch were grouped based on the particular types of pollutant pulses. Samples that contained more Na^+^, Ba^2+^, Mg^2+^, Cu, Sr^2+^, Ca^2+^, and K^+^ tended to be associated with road salt (red and blue symbols) or wastewater treatment (purple symbols; positive PC1) (*sensu* Amrhein et al., 1992; Acosta et al., 2011; Galella et al., 2021). Samples that contained less Na^+^, Ba^2+^, Mg^2+^, Cu, Sr^2+^, Ca^2+^, and K^+^ tended to be associated with stream baseflow conditions (pink and green symbols) or stormwater runoff (yellow symbols; negative PC1). Relative to road salt pulses, wastewater pulses were less enriched in Fe and more enriched with Ca^2+^, Sr^2+^, and K^+^ (purple symbols shifted down towards negative PC2). Summer baseflow samples were enriched in Fe relative to winter baseflow samples, perhaps because Fe was mobilized in the summer during low oxygen conditions (note absence of pink symbols from positive PC2).

### 3.3 Chemical cocktails form longitudinally along watershed flowpaths

**FIGURE 5:**
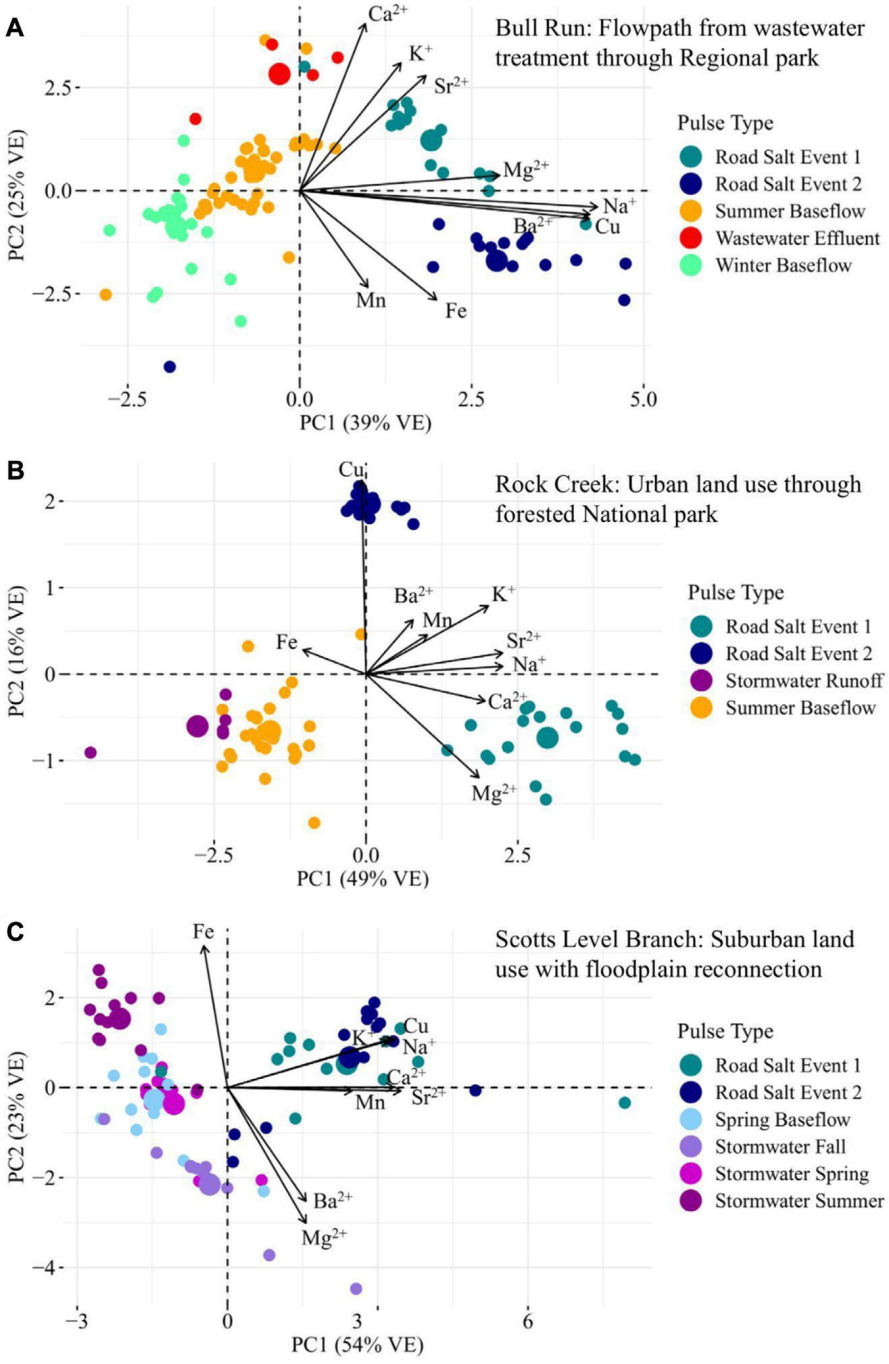
Principal component analysis of each stream sampled for longitudinal monitoring along flowpaths. VE indicates the amount of variance explained by each principal component. The three streams which were monitored longitudinally are analyzed here. In all three synoptics, the road salt chemical cocktails occur on the right side of the biplot. Stormwater and baseflow synoptics are on the left side of the plot, which were less enriched with salt ions.

Because each stream studied had different longitudinal flowpath lengths, sampling conditions, and surrounding watershed land uses, different chemical cocktails were sometimes evident for different streams (Bernier, 1985). We present a few examples of this below, drawing on the results from three of the streams we sampled: Bull Run, Rock Creek, and Scotts Level Branch. In all streams we evaluated, road salting events may have changed the chemical cocktails that were present. This is evident in the strong loading of samples from road salt events on PC1 (the dominant pattern in ion composition) across all panels of Figure 5 (see Supplementary Table S3 for the discriminant matrix). This said, different chemical cocktails can form across road salt events within the same stream. Baseflow and stormflow chemical cocktails tended to have lower concentrations of salt ions and trace metals than other pulse types. Their composition varies by stream and possibly is a function of impervious surface cover.

#### 3.3.1 Bull Run: chemical cocktails form along flowpath from wastewater treatment plant through regional parks

In Bull Run, different road salting events along the flowpath exhibit different distinct chemical cocktails. Both are elevated in salt ions, however, road salt event 1 was more influenced by Mg^2+^ and Sr^2+^ (Mg^2+^ mobilizes Sr^2+^) and road salt two was influenced by Na^+^, Cu, and Ba^2+^ (Na^+^ mobilizes Cu) (Galella et al., 2021).

**FIGURE 6:**
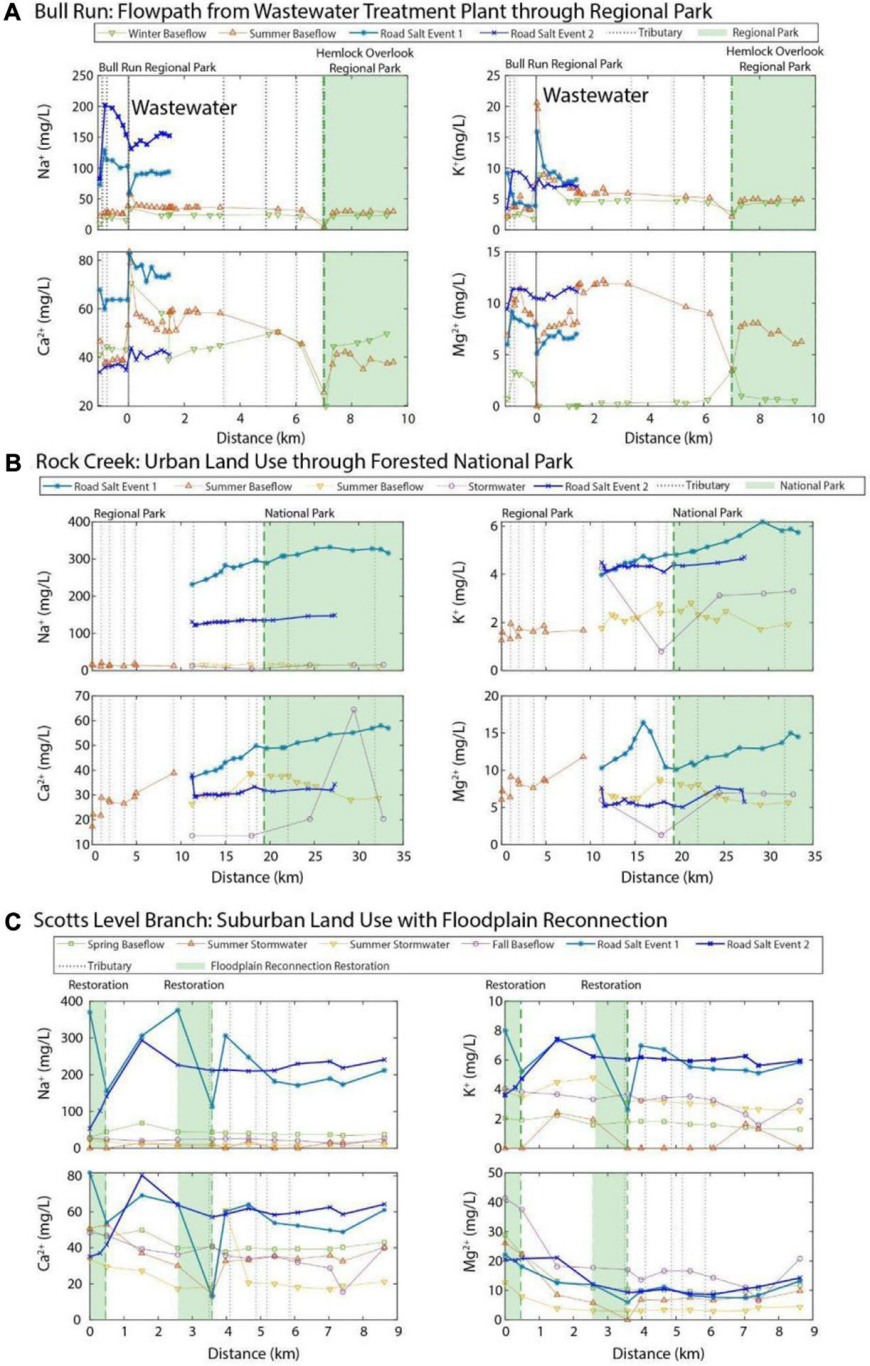
Longitudinal plots of the base cation concentrations vs. distance downstream at the three spatial sampling sites. The green dashed lines indicate either restoration or the transition to conservation. The solid black line in 7a is the wastewater treatment plant effluent which discharges into Bull Run. The lack of attenuation of salt ion concentrations within Rock Creek National Park is from storm drains, leaky sewer pipes, and combined sewer sanitary flow entering the stream during rainfall runoff events.

Water samples collected near and upstream to the wastewater treatment plant have a chemical cocktail characterized by high Ca^2+^, K^+^, and Sr^2+^ and low Mn and Fe (blue symbols; positive PC2, Figure 5A). While wastewater effluent can act as a source of Na^+^ (Bhide et al., 2021), individual road salt events increase Na^+^ concentrations in the stream more than wastewater does. This chemical signature aligns with the longitudinal trends in Figure 6—at the wastewater treatment plant, the in-stream concentrations of K^+^ increased in the stream by an average of 228% and the in-stream Ca^2+^ concentrations increased by an average of 44%. This makes Ca^2+^, K^+^, and Sr^2+^ the chemical signature of ions from the wastewater treatment effluent in Bull Run (Jalali et al., 2008). The baseflow samples had low concentrations of all salt ions and trace metals measured. The summer baseflow samples had concentrations that were influenced by the wastewater treatment plant.

#### 3.3.2 Rock Creek: chemical cocktails form along flowpath from urban land use through forested national park

A stronger divergence between the two road salt events is evident along the flowpath from Rock Creek Regional Park to Rock Creek National Park. The chemical cocktails from road salt event 1 had high concentrations of salt ions, particularly Na^+^, Ca^2+^, and Mg^2+^, whereas road salt event 2 had higher concentrations of Cu (Figure 5B). These differences are likely due to the conditions during the road salting event. During that snow event, the stream’s conductivity at the USGS gauge in Rock Creek National Park peaked around 11,700 μS/cm with a discharge of 1.9 ft^3^/s (Supplementary Figure S3) (peak specific conductance of 6,230 μS/cm, discharge of 10.5 ft^3^/s), which may have transmitted salt ions (K^+^, Na^+^, Ca^2+^, Mg^2+^) out of the watershed more quickly. During baseflow, Rock Creek exhibited stormwater and baseflow samples with elevated ion concentrations, in particular Fe (Figure 5B).

#### 3.3.3 Scotts Level Branch: chemical cocktails form along flowpath along suburban land use with floodplain reconnection

The composition of the chemical cocktails formed during baseflow and storms depended on the impervious surface cover in the watershed. Scotts Level Branch (Figure 5C) has the highest impervious surface cover as well as the smallest watershed area. The stormwater and baseflow events grouped on the biplot by the seasons, though all were elevated in salt ions. In contrast to the other two streams, both road salt events at Scotts Level Branch were associated with similar chemical cocktails, containing high concentrations of a highly correlated chemical cocktail comprised of Cu, Na^+^, K^+^, Ca^2+^, Mn, and Sr^2+^ (Figure 6C). The summer stormwater and summer baseflow events were correlated with chemical cocktails heavily enriched in Fe. During the storm events in the spring and the fall, the chemical cocktail was enriched in both salt ions and Fe.

### 3.4 Attenuation of chemical cocktails across different hydrologic conditions

Chemical cocktails are formed and can be attenuated along flowpaths (see Figure 6), but these patterns depend on dominant pollution type (*e.g.*, from road salt events, wastewater discharge, baseflow, etc.), season, land use, and drainage area. We discuss a few examples of patterns of longitudinal attenuation of chemical cocktails below.

#### 3.4.1 Chemical cocktails are attenuated along flowpaths through riparian buffers and regional parks

Wastewater effluent transiently increased concentrations of Ca^2+^, K^+^, and Na^+^ in spring and summer baseflow at Bull Run. Concentrations of these ions subsequently declined along Bull Run’s flowpath, the majority of which is located within a riparian buffer zone that ultimately feeds into Hemlock Overlook Regional Park (green region, Figure 6A). Longitudinal patterns in Mg^2+^ concentrations were somewhat different (*i.e.*, they were lower in wastewater effluent than the uppermost regions of the riparian buffer zone, and only began to decline approximately 3.5 km downstream after Little Rocky Run; see dashed line at ~3.5 km, Figure 6A). The rapid decline observed across all salt ions prior to Hemlock Overlook Regional Park reflects the proximity of a large tributary, Popes Head Run (see leftmost green dashed line, Figure 6A). Approximately 86% of the Popes Head Run drainage area was classified as residential-conservation in 1982 to preserve water quality in the Occoquan Reservoir (Fairfax County, 2005).

For both road salt events, Na^+^ and Mg^2+^ peaked higher up in the watershed (*i.e.*, at the confluence of Cub Run and Bull Run; leftmost dashed line, Figure 6A). Concentrations of these salt ions declined along the flowpath to the wastewater treatment plant, and then increased somewhat (or stayed relatively stable) thereafter. A similar pattern occurred for K^+^ during the second road salt event, but not the first. During the first road salt event for K^+^ and both road salt events for Ca^2+^, concentrations were highest at the wastewater treatment plant and subsequently stayed stable or declined (*i.e.*, the longitudinal pattern observed was comparable to baseflow conditions).

**FIGURE 7:**
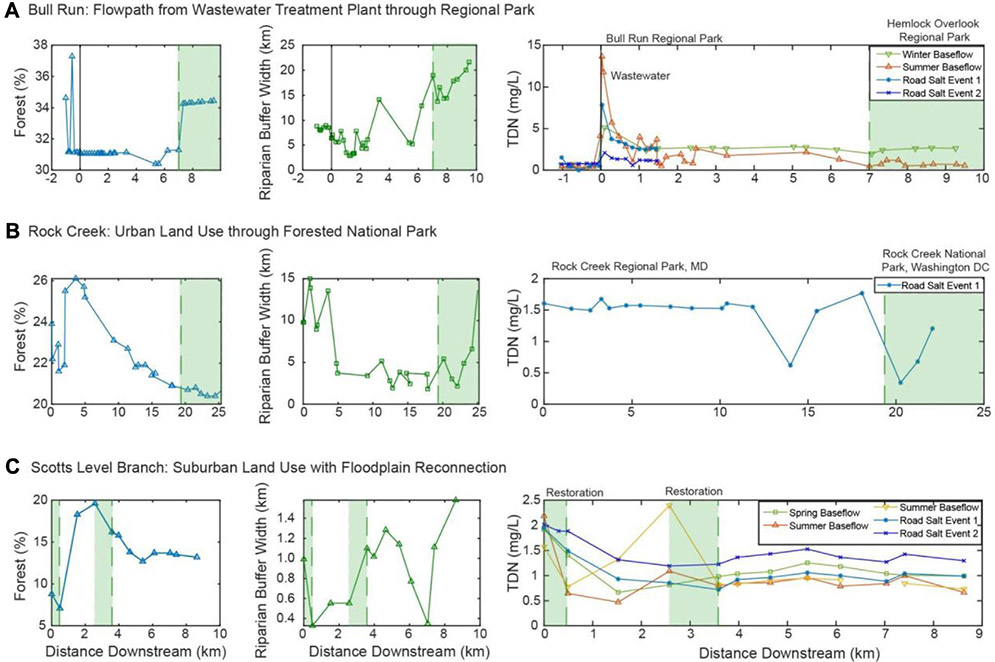
Percent forest within the watershed, riparian buffer width and concentration of total dissolved nitrogen with distance downstream for Bull Run **(A)**, Rock Creek **(B)**, and Scotts Level Branch **(C)**.

**FIGURE 8:**
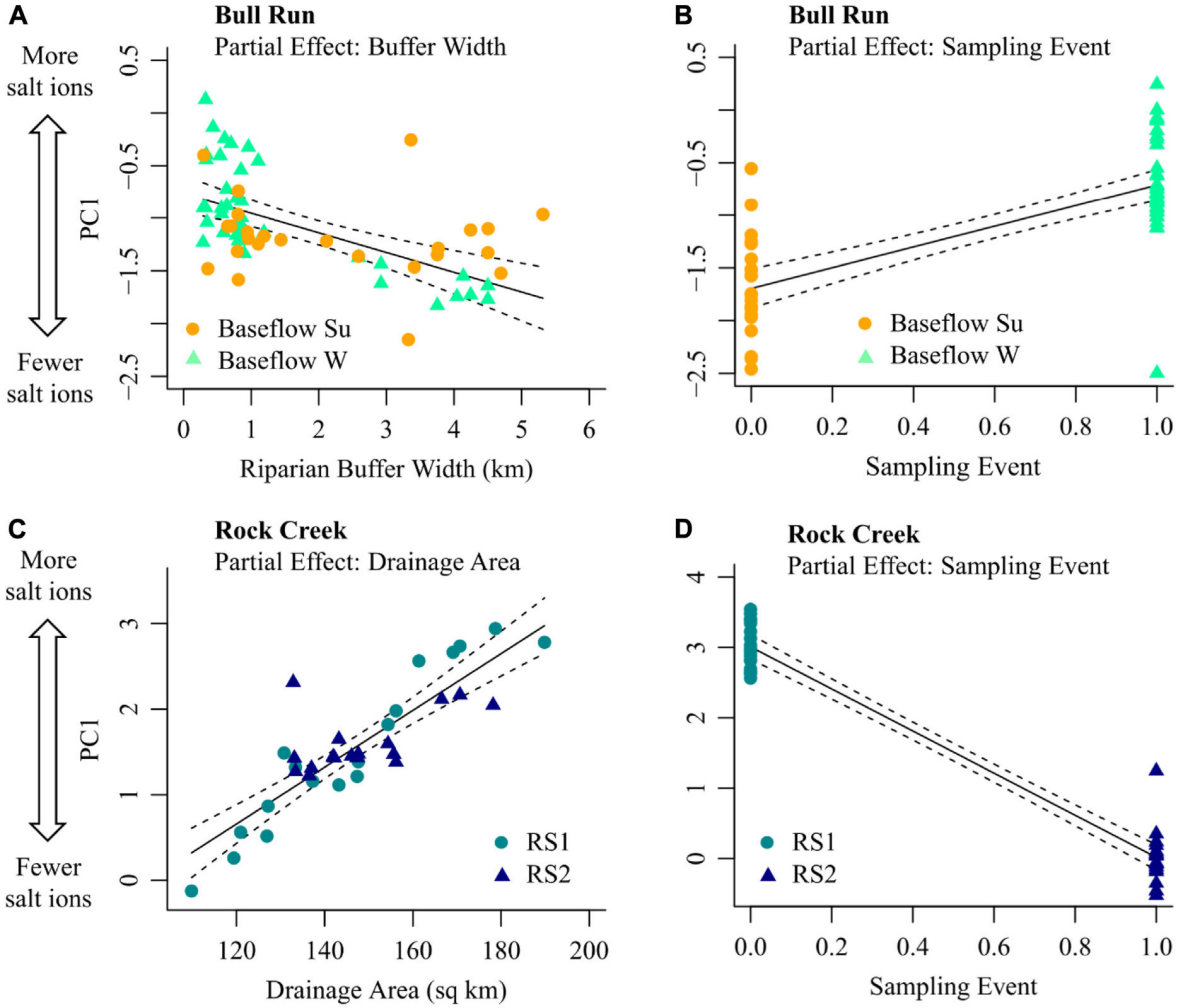
Partial effects plots for each significant variable included in the best-fit multiple linear regression (MLR) models for chemical cocktails at Bull Run **(A,B)** and Rock Creek **(C,D)**. Chemical cocktails are represented using the first principal component of all evaluated ions (*i.e.*, PC1 from Figure 6). The analysis includes only the longest longitudinal synoptics at each site. For Bull Run, these were baseflow synoptics (summer: orange circles; winter: celadon triangles). For Rock Creek, these were road salt synoptics (event 1: turquoise circles, event 2: blue triangles). Solid black lines indicate the partial effect for each significant variable in each best-fit model. Partial effects are evaluated by allowing the independent variable shown on the x-axis of each plot to vary and setting all other independent variables to their mean value. Black dashed lines represent 95% confidence bounds about each partial effect.

The dominant pattern in chemical cocktails for summer and winter baseflow at Bull Run (*i.e.*, PC1 from Figure 5A; positive PC1 indicates higher salt ion concentrations) was well explained by an MLR model including riparian buffer width (ion concentrations were lower when riparian buffer width was higher; see partial effects plot, Figure 7A) and sampling event (ion concentrations were higher in summer baseflow samples than winter baseflow samples; see partial effects plot, Figure 7B). The fitted model explained 66% of the observed variance in PC1 at Bull Run. Approximately two-thirds of this explanatory power was attributable to sampling event (45 of 66%; averaging over ordering, Supplementary Table S4), suggesting that observed chemical cocktails may differ more between summer and winter sampling events (*i.e.*, over time) than along the Bull Run flowpath for a given sampling event. The remainder was attributable to riparian buffer width (21 of 66% variance explained, Supplementary Table S4). Attenuation of chemical cocktails with increasing buffer width was more pronounced in summer than winter baseflow samples (note elevated scatter in winter baseflow samples (celadon points) relative to summer baseflow samples (orange points) in Figure 8A).

#### 3.4.2 Chemical cocktails can be attenuated or enhanced along flowpaths in forested regions that receive multiple storm sewer discharges

In Rock Creek, salt ion concentrations during summer baseflow events tended to increase along the flowpath through the regional park (white region, upper portion of the synoptic) and then decrease through the national park itself (green region, lower portion of the synoptic; Figure 7B). During stormflow and road salt events (purple and blue lines, Figure 6B), ion concentrations tended to increase along the entire flowpath. This was most evident for Ca^2+^, K^+^, and Na^+^. Mg^2+^ was more variable, particularly during road salt event 1. During this event, concentrations of Mg^2+^ increased until approximately 16 km downstream of Rock Creek’s headwaters. Mg^2+^ then rapidly declined over a short ~5 km stretch only to begin increasing again throughout the national park. Changes in discharge and specific conductance were evident throughout each road salt pulse as indicated in Supplementary Figure S4.

The dominant longitudinal pattern in road salt events at Rock Creek (*i.e.*, PC1 from Figure 5B; positive PC1 indicates higher salt ion concentrations) was well explained by an MLR model including watershed drainage area (ion concentrations were higher when drainage area was higher; see partial effects plot, Figure 8C) and sampling event (ion concentrations were higher in the first road salt event than the second; see partial effects plot, Figure 8D). The fitted model explained 95% of the observed variance in PC1 at Rock Creek. As observed with Bull Run, the majority of this explanatory power was attributable to the sampling event (84 of 95%; averaging over ordering, Supplementary Table S5), which suggested that the observed differences in chemical cocktails between road salt events are greater than any longitudinal differences observed within road salt events. The remaining 11% of the explainable variance was captured by drainage area (Supplementary Table S5). This suggests that the increase in salt ion concentrations evident during road salt events across the entire Rock Creek flowpath (recall Figure 7B) may reflect increasing downstream salt inputs from successively larger drainage areas.

#### 3.4.3 Chemical cocktails are attenuated along suburban land use with floodplain reconnection

In Scotts Level Branch, salt ion concentrations during baseflow and stormwater events tended to decrease along the flowpath, leveling out past the second restoration site at approximately 3 km (Figure 6C). This pattern was most notable for divalent base cations (Ca^2+^, Mg^2+^). Different attenuation patterns were observed across all ions (except Mg^2+^) during road salt events. In road salt event 1, salt ion concentrations sharply declined through both restoration regions, but rebounded shortly thereafter, approaching pre-restoration levels (blue curve, Figure 6C). During the second road salt event, salt ion concentrations appear to increase through the first restoration area and decline only marginally through the second. Given that these road salt events occurred within a week of each other, one possible explanation for this pattern is that the second salting event simply overwhelmed restoration areas, and the restoration areas ceased to provide further water quality improvements.

## 4 Discussion

Overall, the formation and attenuation of chemical cocktails formed from freshwater salinization across space and time in watersheds are due to: 1) amounts and types of salt inputs, 2) differences in surrounding land use, conservation, and restoration, and 3) location within the watershed. In the following sections, we discuss patterns in attenuation and dilution of chemical cocktails formed by freshwater salinization, the roles of riparian conservation and restoration areas in attenuation of the chemical cocktails, and the potential for salt to overwhelm dilution and ion exchange processes along flowpaths. We also acknowledge that the dynamics of urban streams are multi-year processes and concentrations are reliant on the streamflow, seasonality, and temporal trends (Corsi et al., 2015).

### 4.1 Formation and attenuation of chemical cocktails downstream

#### 4.1.1 Formation and attenuation of treated wastewater across time and space

Treated wastewater formed a unique, distinct chemical cocktail of Ca^2+^, K^+^, and Sr^2+^ (Figure 5A) in this study. During baseflow events, the concentrations of Na^+^, Ca^2+^, and K^+^ increased at the wastewater treatment plant while Mg^2+^ declined (Figure 7A). Some common elements and ions in wastewater are total nitrogen, K^+^, Na^+^, Cl^−^, and SO_4_^−2^ with Mn, Ni^2+^, Zn^2+^, Cr, Cu, As, Pb, or Cd^2+^ (Kaushal et al., 2020). While Na^+^ is typically a dominant ion in treated wastewater effluent, the excess of Na^+^ during the road salt events causes Ca^2+^ and K^+^ to be a unique signature from the effluent. Although not measured in this study, wastewater is a source of other nutrients, organic matter, emergent pollutants, and pathogens to streams (Castelar et al., 2022), all of which can create chemical cocktails that can be transported and transformed along a flowpath.

Bull Run receives the highly treated wastewater from the wastewater treatment facility and is a part of the indirect potable reuse (IPR) management strategy. Indirect potable reuse is a sustainable management practice that discharges highly treated wastewater to the surface water and the groundwater that can, in turn, be used as a drinking water source for a community downstream (Rodriguez et al., 2009). The wastewater treatment facility, UOSA, discharges effluent into Bull Run as surface water recharge which flows to the Occoquan Reservoir, the receiving waters for the Fairfax Country Griffith drinking water facility (Onyango et al., 2014). Therefore, the watershed management system is established upon the ability for Bull Run to attenuate the additional nutrients and ions, particularly total dissolved nitrogen.

Salts from the untreated wastewater are not removed and can be compounded from wastewater treatment plant and directly discharged into the stream during a road salt event (Bhide et al., 2021). During dry and median weather conditions, the treated wastewater effluent is the majority of the sodium mass load that enters the reservoir (Bhide et al., 2021). The sodium mass loadings from the wastewater treatment facility to Bull Run can vary from 60% to 80% in dry weather conditions (Bhide et al., 2021). This is shown in the PCA—the summer baseflow chemical cocktails found at Bull Run were most similar to wastewater effluent during the low flow conditions, indicating that the wastewater treatment facility was a large contributor to Bull Run’s discharge. The capacity to dilute, biogeochemically process, and attenuate these nutrients and ions from the wastewater treatment plant along a stream’s flowpath is dependent on the flow conditions, residence times of the ions, ion exchange capacity of sediments, and seasonality (Castelar et al., 2022; Kaushal et al., 2022). Depending on the climatic conditions and the discharge, Bull Run can act as a source, sink, or transporter of ions downstream of the wastewater treatment plant. Future work should continue to investigate the transport and biogeochemical transformation of various pollutants and contaminants before and after wastewater effluent enters streams.

#### 4.1.2 Formation and attenuation of road salt chemical cocktails along flowpaths

Differences among the formation of salt ion-enriched chemical cocktails from road salt events could depend on the timing and intensity (*i.e.*, the quantity of road salt application per impervious surface cover) of road salt application, the surrounding land use, and the timing of the sampling event within the watershed. We observed trace metal comobilization with base cations during road salt events both temporally and longitudinally, potentially due to cation exchange (Figure 7) (Kaushal et al., 2019; Galella et al., 2021; Kaushal et al., 2021). As the specific conductance rises and peaks from the influx of road salt, there is a strong relationship between the base cations and the trace metals (Galella et al., 2021). After the initial peak in road salt pulse, the declining specific conductance suggests a weaker relationship between the base cations and trace metals (Galella et al., 2021). However, the relationships between chemical cocktails downstream may be due to varying watershed characteristics such as the surrounding land use and the management strategies implemented into the watershed (Kaushal et al., 2023a).

There were not consistent longitudinal downstream patterns of dilution or attenuation along the flowpath during road salt events, even within conservation and restoration areas. Through all the management areas (*i.e.*, Bull Run Regional Park, Rock Creek Regional and National Park, and the restoration sites along Scotts Level Branch) there were variable patterns of retention and release with Na^+^, Ca^2+^, Mg^2+^, and K^+^ (Figure 6). These variable patterns are potentially due to the hydrologic connectivity of the stream to surrounding land use through subsurface storm drains and exposed sewer lines.

The surrounding watershed land use around Bull Run has been managed and regulated since the early 1970s (there is newer development in northern Virginia compared with our urban Maryland sites). Fairfax County in Virginia has required stormwater ponds as a management strategy to protect the drinking water reservoir downstream (Fairfax County, 2023). The Cub Run tributary and Bull Run watersheds have over 420 stormwater ponds and 26% of the watershed area has been conserved and restored to improve the peak flows and pollution runoff (Fairfax County, 2023). Therefore, there are more measures to reduce stormwater and road runoff pollution implemented in Bull Run than Rock Creek, leading to differences amongst the patterns during road salt events.

Around Rock Creek, densely urban Washington D.C. surrounds Rock Creek National Park. Although the riparian buffer and forest cover increase within the park, the drainage area of the watershed and the sampling event (Figures 8C, D) were the parameters that best explained the chemical cocktails captured by PC1. The PCA in Figure 5B shows that the two road salt events had different chemical cocktails, with the first road salt event in the positive PC1 and negative PC2 space (higher concentration of salt ions, lower concentration of redox sensitive elements) and road salt event 2 tracking with the positive PC2 space (higher concentration of redox sensitive elements). The partial effect of higher drainage areas with more salt ions indicates the influence of the sewer lines and storm drains entering the stream, with some as combined storm and sanitary sewers that were built in the 1880s (Hartman, 2001). During rainfall or snowfall runoff events, some of the storm and wastewater flows to a wastewater treatment plant in the region but the remaining untreated combined sewage overflow is discharged into streams directly, including about 4 million gallons that empty into Rock Creek (Hartman, 2001; Groves and Wolfe, 2019).

### 4.3 Formation and attenuation of chemical cocktails from stormwater runoff across time and space

Stormwater runoff and baseflow conditions formed chemical cocktails less enriched in salt ions and trace metals with respect to road salt chemical cocktails. The stormwater runoff events are generally shorter in length than winter stormwater events (Hallberg et al., 2007). Many of the samples analyzed above were collected on the receding limb of the hydrograph a few days after a stormwater event and therefore not capturing the “first flush” of pollutants and heat from impervious surfaces (Kayhanian et al., 2012).

There are longitudinal and temporal patterns which emerged during baseflow and stormflow events as shown by the partial effects plots in Figure 8. The Bull Run baseflow events had a lower concentration in salt ions than the road salt events. The MLR analysis for Bull Run during the summer and winter baseflow events showed that the two significant predictors of PC1 are the buffer width and the sampling event (Figures 8A, B). Figure 8A suggests that, controlling for the sampling event (winter vs. summer), an increase in buffer width results in fewer salt ions. In the summer and early fall, there are warmer surface water temperatures, more direct sunlight, and higher autotrophic uptake in the stream causing higher primary productivity (Ledford et al., 2017). Therefore, there is the potential for attenuation of salt ions with increased riparian buffer width. Figure 8B suggests that, controlling for riparian buffer width, the summer baseflow sampling event had a higher concentration of salt ions than the winter baseflow event. Recent work suggests there can be retention of the salt ions along floodplains in soils across the year (Ledford et al., 2016; Maas et al., 2021). Salt ions accumulate in the sediments and groundwater, resulting in elevated salt ion and trace metal concentrations several months after the specific conductance and Na^+^ concentration decline (Cooper et al., 2014; Cockerill et al., 2017; Kaushal et al., 2019; Galella et al., 2021). Chloride is primarily stored within the saturated zone of riparian floodplains in soils, groundwater, or other subsurface storage (Kelly et al., 2008; Ledford et al., 2016). Groundwater recharge during overbank flood events in the winter promotes the storage of chloride and other ions within the floodplain (Ledford et al., 2016). During low chloride overbank flood events, the salty groundwater is flushed out of the floodplains and into the stream, acting as a source of salt ions (Ledford et al., 2016; Cockerill et al., 2017).

At Rock Creek and Scotts Level Branch, the summer baseflow and stormwater runoff both had higher concentrations of Fe than during road salt conditions. Potential Fe sources associated with increases in dissolved organic matter and reducing conditions in the watershed (Figures 5B, C) (Ekström et al., 2016). Chemical cocktails in stormwater runoff are more challenging to quantify and characterize due to the diffuse sources, including soil erosion, vehicles, human and animal waste, fertilizers, household chemicals, industrial processes, atmospheric fallout, and paints and preservatives (Aryal et al., 2010). Common inorganic ions in urban stormwater runoff include heavy metals, such as Pb, Cr, Zn^2+^, Cu, Ni^2+^, and Cd^2+^ generally associated with finer sediment fraction runoff (Aryal et al., 2010).

### 4.4 Roles of conservation and restoration in the attenuation of chemical cocktails, salt saturation, and limitations in response to freshwater salinization syndrome (FSS)

Conservation and restoration areas are implemented in impaired watersheds to reduce the concentration of nutrients such as nitrogen through denitrification (Mayer et al., 2007). However, these areas have the potential to attenuate transport of salt pollution further downstream when the rates of watershed salt loading are lower such as during baseflow and stormflow events, however, the effects are variable during road salt events. There were patterns of increases, plateaus, and declines along a flowpath, depending on the surrounding watershed characteristics. There were patterns of increase with all the salt ions in parks at Bull Run and Rock Creek (Figures 6A, B). In the floodplain reconnection restoration at Scotts Level Branch, the restoration acted as a sink during the first road salt event and a source of ions during the second road salt event (Figure 6C). These green spaces, such as urban forests and restorations, benefit urban streams by reducing pollutant concentrations in urban runoff (Livesley et al., 2016). In comparison to impervious surfaces, trees and vegetated areas can intercept more rainfall, have higher infiltration rates into soils, and reduce runoff during stormwater events (Nowak, 2006).

Previous research suggested nitrogen can be effectively removed by riparian buffers (Mayer et al., 2007). Wider riparian buffers provide more area for root uptake of nitrogen and favor denitrification (Mayer et al., 2007). At Bull Run along the flowpath from the wastewater treatment plant through the regional parks, there were no significant patterns between riparian buffer width and total dissolved nitrogen concentrations during the summer or winter baseflow (Figure 7A). This could be due to the vegetation type, root zone depth, or hydrological flowpath (Mayer et al., 2007). However, during baseflow events, there are some significant patterns of decreasing ion concentration with increased riparian buffer width. In the summer and early fall, there are warmer surface water temperatures, more direct sunlight, and higher autotrophic uptake in the stream causing higher primary productivity (Ledford et al., 2017). Therefore, there is the potential for attenuation of salt ions with increased riparian buffer width. However, during road salt events, there are positive relationships. At Rock Creek, the riparian buffer width increases at the end of the flowpath in Washington D.C. (Figure 7B). Many of the ions increased with increasing riparian buffer width, indicating that the riparian buffer was overwhelmed from freshwater salinization ions.

## 5 Knowledge gaps and future research

Restoration and conservation areas are often implemented for their ability to reduce denitrification and improve stream quality. However, these sites are usually studied on a small scale for a relatively short time period and analyzes only a few elements or nutrients at a time. Here, we studied salt ions, trace metals, and nutrients across time and space before, during, and after freshwater salinization pollution events. By temporally and spatially studying chemical cocktails along restoration and conservation flowpaths, rather than individual contaminants, there can be improvements to pollutant tracking, the co-management of pollutants, the determination of thresholds for contaminant mobilization in response to freshwater salinization, and the development of sensor proxies for harmful pollutants (Trowbridge et al., 2010; Moore et al., 2019; Kaushal et al., 2020; Galella et al., 2021). These methodologies can highlight areas with increased influence of FSS and establish the locations to effectively implement restoration and conservation (Kaushal et al., 2022; 2023b).

In recent years, there has been an emphasis on understanding the retention and release of salt ions from stormwater best management practices such as retention ponds, rain gardens, natural or accidental wetlands, and bioswales (Maas et al., 2021; Kaushal et al., 2022). However, less research has focused on stream restoration efforts such as floodplain reconnection to understand if there is retention of salt ions in the floodplain soils during various freshwater salinization pollution events. For example, if the concentrations of chemical cocktails decrease downstream of restored stream reaches and floodplains and riparian buffers, these features can be used to manage different types of chemical cocktails in the future.

The chemical cocktail methodology provides a statistical way to assess the sources, transport, and transformation in elemental combinations over space and time (Kaushal et al., 2018a; Kaushal et al., 2020; Morel et al., 2020; Galella et al., 2021). Other studies can use the chemical cocktail approach and can expand the network of data to include a variety of different geographical locations, pollution events, and locations. This work illustrates how chemical cocktails can be parsed from complex environmental data using PCA. PCA results characterize chemical cocktails in a way that is reproducible. For example, every PCA analysis is associated with a series of reproducible equations. Based on these equations, other studies can compare the chemical cocktails measured in their own regions of the world to the chemical cocktails we have characterized using the equations. Thus, our approach offers a unique point of comparison for other studies looking to evaluate chemical cocktails in their own systems. In addition, multivariate statistical approaches may eventually facilitate quantitative tracking of chemical cocktails from different pollution sources using end members and mixing models.

## 6 Conclusion

Overall, the formation and attenuation of chemical cocktails formed from freshwater salinization across space and time in watersheds are due to: 1) amounts and types of salt inputs, 2) differences in surrounding land use, conservation, and restoration, and 3) location within the watershed. FSS manifests itself in diverse forms and can mobilize different chemical cocktails based on pollution sources (*e.g.*, sewage, urban runoff, and road salt). Chemical cocktails show different patterns of attenuation downstream based on pollution sources, and we found that there can be the potential for conservation and restoration areas to reduce FSS.

Three categories of chemical cocktails formed over space and time: 1) higher concentration of salt ion and trace metals chemical cocktails from road salt events, 2) chemical cocktails lower in salt ions and trace metals during baseflow and stormwater events, and 3) Ca^2+^ and K^+^ enriched chemical cocktails from wastewater effluent. In general, chemical cocktails during baseflow and stormwater events were attenuated downstream over short time scales as streams flowed through restoration and conservation areas. However, road salt events overwhelmed the capacity for restoration and conservation to attenuate chemical cocktails. Few studies have focused on the transport and transformation of salt ions, metals, and nutrients along watershed flowpaths, particularly flowpaths with conservation and restoration. Our results provide further insights on the formation and potential for longitudinal attenuation of chemical cocktails during different pollution events and within reaches with restoration and conservation. Conservation and restoration can improve downstream water quality associated with different pollution events, but the attenuation capacity of streams, floodplains, and riparian zones can be overwhelmed by freshwater salinization.

## Supplementary Material

Supplement1

## Figures and Tables

**TABLE 1:** Watershed and long-term monitoring sensor characteristics for each stream. The latitude and longitude and the watershed characteristics for the temporal monitoring sites, the Anacostia at Bladensburg, Paint Branch, and Sligo Creek, are from the USGS gauge station. The latitude and longitude and the watershed characteristics for the spatial monitoring sites, Scotts Level Branch, Bull Run, and Rock Creek, are from the furthest downstream synoptic sampling point. Scotts Level Branch was monitored both temporally and spatially. All sites have specific conductivity and discharge high-frequency sensors except the Anacostia at Bladensburg, which does not have discharge.

Sites	Monitoring station	Latitude, longitude	Drainage area (km^2^)	Forest cover (%)	Impervious surface cover (%)	Agricultural cover (%)	Start of long term record
Anacostia at Bladensburg, MD	USGS 01651007	38.93424, −76.93923	331.5	24.3	30.9	6.0	July 2021
Paint Branch, MD	USGS 01649190	39.02972, −76.95067	40.66	27	30.8	4.6	October 2007
Sligo Creek, MD	UGSG 01650800	38.98605, −77.00431	16.76	11.9	41.4	0.04	October 2008
Scotts Level Branch, MD	USGS 01589290	39.36058, −76.74620	10.41	13.2	39.3	3.3	October 2005
Bull Run, VA	Virginia Tech’s Occoquan Monitoring Lab	38.72412, −77.38027	502.46	35.67	13	12.6	1972
Rock Creek, MD and DC	USGS 01648010	38.90008, −77.05738	198.14	21.5	31.4	6.2	April 2007

**TABLE 2:** Description of each spatial sampling flowpath.

Stream	Date	Longitudinal distance (km)	Number of samples collected	Median distance between sampling sites (km)	Pollution pulse captured
Bull Run	13 January 2021	16.4	41	0.5	Baseflow; denitrification in WWTP; no road salt
	17 September 2021	15.8	68	0.2	Baseflow; rained while sampling; reduced denitrification in WWTP
	8 January 2022	2.4	20	0.2	Road salting event 1; reduced denitrification in WWTP
	17 January 2022	2.4	20	0.2	Road salting event 2; reduced denitrification in WWTP
Rock Creek	6 February 2021	21.2	25	1.2	Road salting event 1
	9 August 2021	9.2	15	1.0	Summer baseflow
	13 August 2021	20.9	20	1.0	Summer baseflow
	3 September 2021	21.5	5	4.3	Urban stormwater; after tropical storm Ida
	19 January 2022	16	25	0.6	Road salting event 2
Scotts Level Branch	27 March 2021	8.6	12	0.8	Spring baseflow
	12 August 2021	8.6	12	0.8	Summer stormwater
	2 September 2021	8.6	12	0.8	Summer stormwater; after tropical storm Ida
	2 November 2021	8.6	12	0.8	Fall baseflow
	5 January 2022	8.6	12	0.8	Road salting event 1
	12 January 2022	8.6	13	0.7	Road salting event 2

## Data Availability

The raw data supporting the conclusion of this article will be made available by the authors, without undue reservation.

## Footnotes

1 Throughout this paper, we will indicate charges on elements with only one oxidation state; if there are multiple oxidation states, we will give the abbreviation of the element.
