# Supplementary material for: Freshwater salinization syndrome limits management efforts to improve water quality
Source: Front Environ Sci. Author manuscript; Available in PMC 2024 Sep 22. (PMC10568995; doi:10.3389/fenvs.2023.1106581)
Supplement: Supplement1 [file NIHMS1933691-supplement-Supplement1.pdf]

Supplementary Material

Freshwater Salinization Syndrome limits management efforts to improve water quality

Carly M. Maas*, Sujay S. Kaushal, Megan A. Rippy, Paul M. Mayer, Stanley B. Grant, Ruth R. Shatkay, Joseph T. Malin, Shantanu V. Bhide, Peter Vikesland, Lauren Krauss, Jenna E. Reimer, and Alexis M. Yaculak

*** Correspondence:** Corresponding Author: maascm@umd.edu

Supplementary Tables

SI Table 1. Limits of detection for each element analyzed in this study.

Ion	Detection Limit	Ion	Detection Limit
TDN	5 µg/L	K^+	0.4 µg/L
B	0.2 µg/L	Mg^{2+}	0.005 µg/L
Ba^{2+}	0.01 µg/L	Mn	0.03 µg/L
Ca^{2+}	0.005 µg/L	Na^+	0.3 µg/L
Cu	0.4 µg/L	S^{2-}	5 µg/L
Fe	0.1 µg/L	Sr^{2+}	0.007 µg/L

SI Table 2. Discriminant matrix for Figure 4 for applications of the principal component analysis.

	Figure 5a: Temporal Monitoring				***Figure 5b: Spatial Monitoring***			
**Ion**	**Mean**	**SD**	**PC1**	**PC2**	**Mean**	**SD**	**PC1**	**PC2**
**Ba^{2+}**	0.18	0.07	-0.02	0.12	0.12	0.069	0.31	0.15
**Ca^{2+}**	31.0	13.76	0.44	-0.11	43.45	20.65	0.43	-0.26
**Cu**	0.02	0.03	0.11	0.21	0.039	0.46	0.32	0.040
**Fe**	0.14	0.10	-0.17	0.53	0.16	0.17	-0.062	0.64
**K^{+}**	3.68	1.23	0.41	0.22	4.49	2.99	0.33	-0.31
**Mg^{2+}**	7.51	3.59	-0.30	-0.21	9.09	6.12	0.32	0.098
**Mn**	0.04	0.05	0.11	0.60	0.083	0.13	0.23	0.57
**Na^{+}**	48.78	59.34	0.32	0.39	84.62	103.37	0.41	0.22
**Sr^{2+}**	0.16	0.07	0.47	-0.10	0.18	0.092	0.43	-0.14
**TDN**	0.96	0.31	0.41	-0.14				

SI Table 3. Discriminant matrix for Figure 5 for applications of the principal component analysis.

	Figure 6A: Bull Run				*Figure 6B: Rock Creek*				*Figure 6C: Scotts Level Branch*			
**Ion**	**Mean**	**SD**	**PC1**	**PC2**	**Mean**	**SD**	**PC1**	**PC2**	**Mean**	**SD**	**PC1**	**PC2**
**Ba^{2+}**	0.091	0.045	0.49	-0.084	0.12	0.069	0.31	0.15	0.15	0.089	0.19	-0.47
**Ca^{2+}**	50.75	15.65	0.11	0.59	43.45	20.65	0.43	-0.26	43.17	15.86	0.41	0.0017
**Cu**	0.042	0.036	0.49	-0.099	0.039	0.46	0.32	0.040	0.044	0.055	0.40	0.20
**Fe**	0.087	0.074	0.23	-0.38	0.16	0.17	-0.062	0.64	0.13	0.088	-0.058	0.59
**K^{+}**	6.40	3.66	0.17	0.45	4.49	2.99	0.33	-0.31	3.63	1.93	0.39	0.19
**Mg^{2+}**	6.55	4.12	0.34	0.054	9.09	6.12	0.32	0.098	11.96	7.58	0.19	-0.56
**Mn**	0.039	0.065	0.11	-0.34	0.083	0.13	0.23	0.57	0.058	0.053	0.30	-0.013
**Na^{+}**	57.76	48.48	0.50	-0.058	84.62	103.37	0.41	0.22	88.32	102.98	0.41	0.20
**Sr^{2+}**	0.21	0.057	0.21	0.41	0.18	0.092	0.43	-0.14	0.17	0.091	0.42	-0.013

SI Table 4: Candidate Regression Models Explaining Variability in Chemical Cocktails at Bull Run (Baseflow)

MODELS	Parameters	Coefficients (SE)[1]	LMG[2]	BIC[3]	w_{BIC}[4]	RMSE (LOOCV)[5]	RMSE (tot)[6]	R^2 (tot)[7]
**Model 1 (best-fit)**	Intercept*	-1.37 (0.13)	--	93.9	0.69	0.34	0.44	0.66
	Bw[a] (km)*	-0.19 (0.04)	0.21					
	Event*	0.98 (0.12)	0.45					
**Model 2**	Intercept•	-2.39 (0.67)	--	95.6	0.3	0.34	0.43	0.68
	DA[b] (km^2)	0.003 (0.002)	0.07					
	Bw (km)*	-0.27 (0.07)	0.17					
	Event*	0.98 (0.12)	0.44					

*: significant at the $p < 0.05$ level; •marginally significant at the $p < 0.1$ level;
Abbreviations: [a]BW: riparian buffer width, [b]DA: drainage area
[1]SE: Standard Error; [2]LMG: the contribution of each parameter to overall R^2 (assessed using the averaging over ordering method LMG);
[3]BIC: Bayesian Information Criterion; [4]w_{BIC}: BIC weights;
[5]RMSE (LOOCV): The weighted average Root Mean Squared Error estimated using Leave One Out Cross Validation;
[6]RMSE (tot): Root Mean Squared Error (full model); [7]R^2 (tot): Coefficient of Determination (full model)

SI Table 5: Candidate Regression Models Explaining Variability in Chemical Cocktails at Rock Creek (Deicers)

MODELS	Parameters	Coefficients (SE)[1]	LMG[2]	BIC[3]	w_{BIC}[4]	RMSE (LOOCV)[5]	RMSE (tot)[6]	R^2 (tot)[7]
**Model 1 (best-fit)**	Intercept*	-1.87 (0.51)	--	39.8	0.81	0.29	0.35	0.950
	DA[a] (km^2)*	0.03 (0.003)	0.11					
	Event*	-2.99 (0.12)	0.84					
**Model 2**	Intercept	-0.45 (3.18)	--	42.7	0.19	0.30	0.35	0.950
	DA (km^2)*	0.03 (0.004)	0.11					
	pF[b] (%)	-0.10 (0.14)	0.02					
	Event*	-3.02 (0.13)	0.82					

*: significant at the $p < 0.05$ level; •marginally significant at the $p < 0.1$ level;
Abbreviations: [a]DA: drainage area, [b]pF: percent forest
[1]SE: Standard Error; [2]LMG: the contribution of each parameter to overall R^2 (assessed using the averaging over ordering method LMG);
[3]BIC: Bayesian Information Criterion; [4]w_{BIC}: BIC weights;
[5]RMSE (LOOCV): The weighted average Root Mean Squared Error estimated using Leave One Out Cross Validation;
[6]RMSE (tot): Root Mean Squared Error (full model); [7]R^2 (tot): Coefficient of Determination (full model)

Supplementary Figures

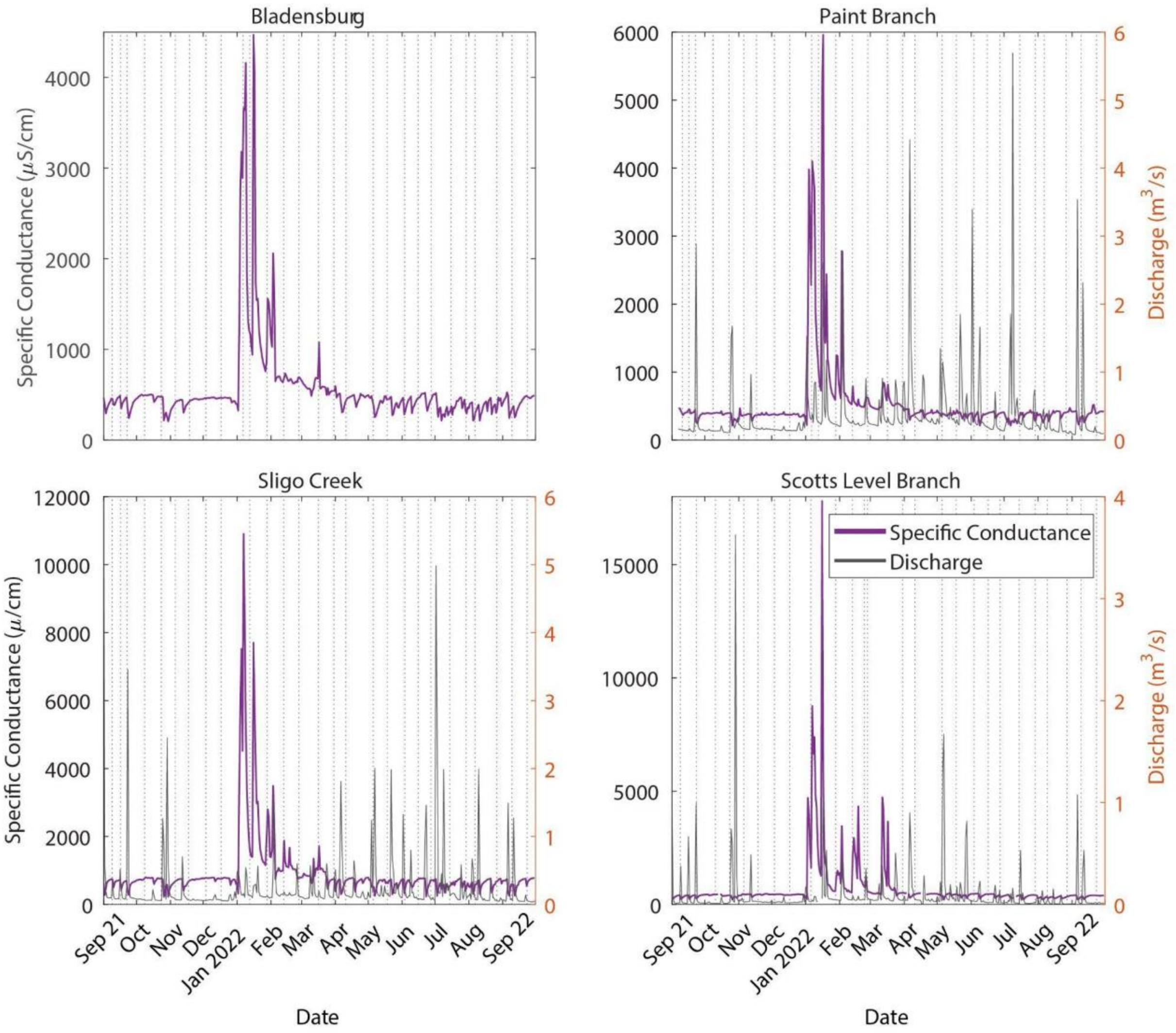

SI Figure 1. Temporal monitoring over an annual cycle from September 1, 2021 to September 30, 2022. The thicker purple lines indicate the specific conductance and the thinner gray lines indicate the discharge. The vertical dashed lines are the dates samples were collected.

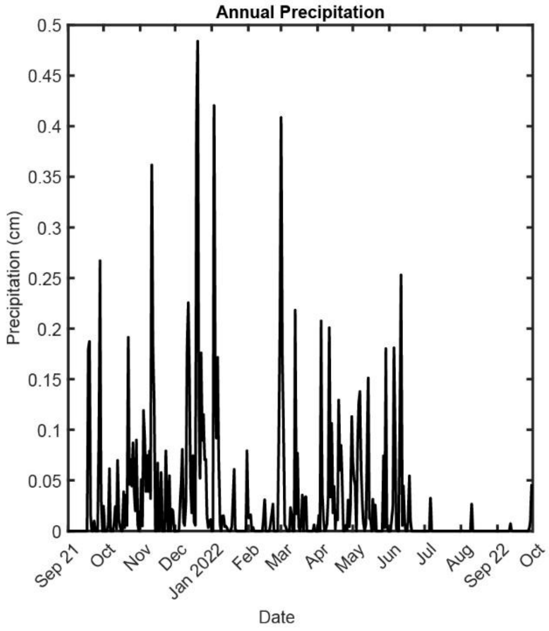

SI Figure 2. The annual precipitation in the Mid-Atlantic region from PRISM data collected at Sligo Creek USGS latitude and longitude from September 1, 2021 to October 1, 2022.

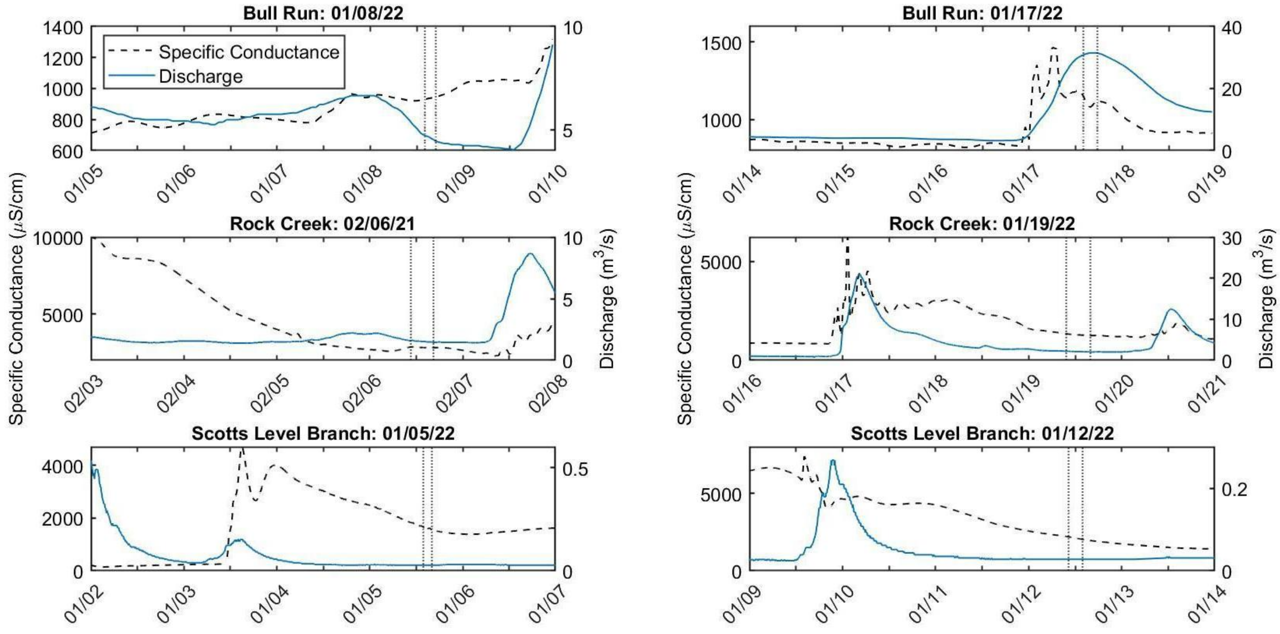

SI Figure 3. Sampling conditions for each of the winter road salt events. Sampling occurred during the dashed vertical lines.

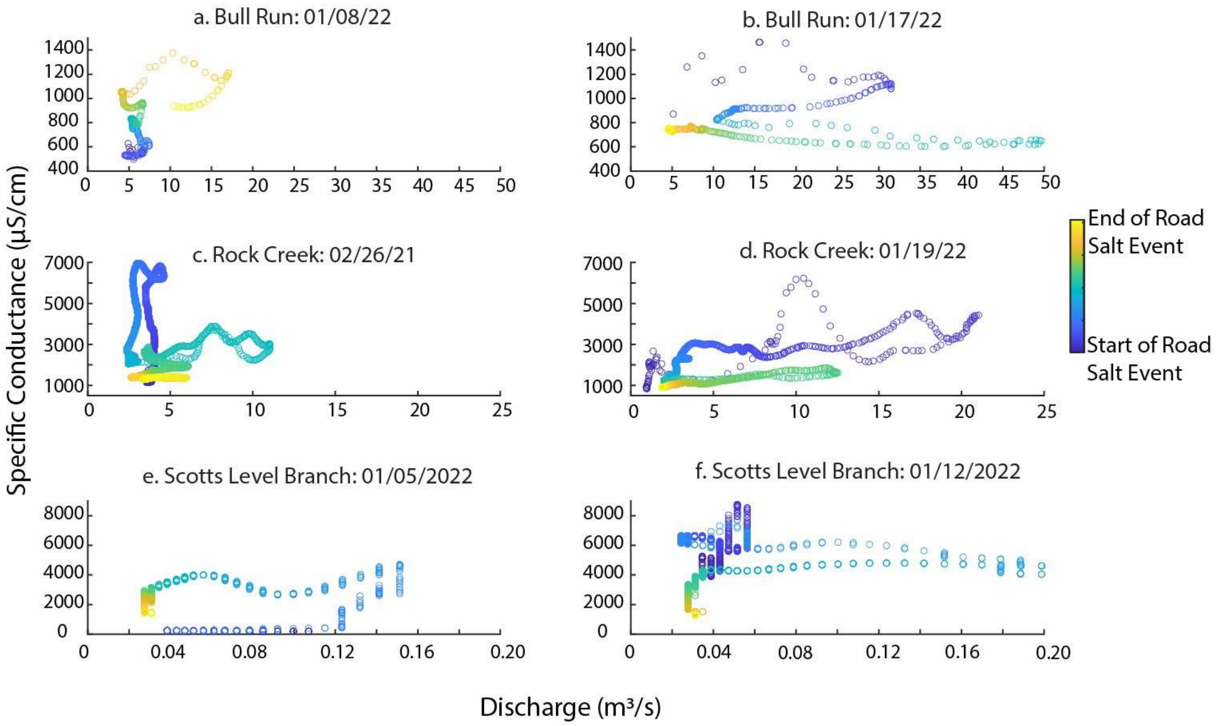

SI Figure 4. Hysteresis loops of discharge and specific conductance from the starting point to the end of the winter road salt events. The data plotted is from the long-term high-frequency monitoring stations along each river. The darker blue circles indicate the start of the road salt event and yellow indicates the end of the road salting events.
